# A Novel Prognostic Index for Metastatic Colon Cancer: The Prognostic Immune Nutritional Index

**DOI:** 10.7759/cureus.33808

**Published:** 2023-01-15

**Authors:** Erkan Kayikcioglu, Gokce Iscan

**Affiliations:** 1 Department of Medical Oncology, Suleyman Demirel University School of Medicine, Isparta, TUR; 2 Department of Family Medicine, Suleyman Demirel University School of Medicine, Isparta, TUR

**Keywords:** prognostic index, prognostic immune nutritional index, metastatic colon cancer, monocyte, albumin

## Abstract

Objective

Systemic inflammation and nutrition are associated with survival outcomes in metastatic colon cancer (mCC) patients. A new and strong prognostic marker named the Prognostic Immune Nutritional Index (PINI) was proposed as the best marker for outcomes in metastatic colon cancer patients. This study aimed to evaluate the prognostic significance of PINI in mCC patients.

Methods

The data of 190 patients who were admitted to our center and diagnosed with mCC between 2010 and 2020 abiding by our inclusion criteria were reviewed retrospectively. Receiver operating characteristic (ROC) analysis was used to identify the optimum cutoff value of PINI for overall survival (OS).

Results

The mean age of the participants was 62.64±11.99 years. The median follow-up time was 25.81 months. According to PINI, the median OS in patients who had PINI<3 was 22.70 months (95% confidence interval (CI): 16.05-29.35), and the median OS in patients who had PINI≥3 was 38.83 months (95% CI: 26.98-37.01) (p<0.001). PINI score lower than 3 was an independent prognostic indicator in multivariate analysis.

Conclusions

PINI was discovered to be an independent prognostic factor in metastatic colorectal cancer. We believe that PINI, which can be calculated using a simple formula, will provide clinicians with important clues when deciding on individual treatment.

## Introduction

Colon cancer is the second most common cause of cancer-related death, with 975,880 deaths annually [1]. Despite chemotherapy and targeted medicines at the advanced stage, the prognosis is dismal. With the introduction of biological agents (cetuximab, panitumumab, and bevacizumab), the median overall survival (OS) reached 28 months in the metastatic stage [2].

Numerous molecular markers and prognostic indicators such as carcinoembryonic antigen (CEA), RAS, BRAF mutation, tumor location, and microsatellite instability (MSI) can be used to determine metastatic colon cancer (mCC) prognosis. RAS and BRAF mutations are not only associated with poor prognosis in colon cancer but also show resistance to anti-epidermal growth factor receptor (EGFR) agents such as cetuximab and panitumumab [3]. Of sporadic colorectal tumors, 10%-15% have MSI caused by mutations in DNA mismatch repair genes such as MLH1, MSH2, MSH6, and PMS2 [4]. In mCC cancer, MSI predicts a favorable prognosis. According to a report, mCC patients with MSI have a survival rate of up to 15% higher than mCC patients with microsatellite stable (MSS) tumors [5]. The left side was associated with a considerably lower risk of death, irrespective of the stage, race, adjuvant chemotherapy, year/number of participants, and study quality, when 66 studies involving more than 1,400,000 people were included in a major systematic review and meta-analysis. A significant predictor of the success of anti-epidermal growth factor receptor (EGFR) therapy in people with metastatic RAS wild-type colon cancer is sidedness [6].

To manage and better direct therapy, develop new therapeutic modalities, and improve existing therapies, clinicians require prognostic and predictive markers. Recent studies focused on how patient immune systems and nutrition affect disease prognosis. The effects of neutrophil/lymphocyte ratio (NLR), platelet/lymphocyte ratio (PLR), monocyte/lymphocyte ratio (MLR), prognostic nutritional index (PNI), systemic inflammatory index (SII), and geriatric nutritional index (GNI) on prognosis were studied in various cancers [7,8]. NLR, an indicator of inflammation and loss of skeletal muscle, and sarcopenia, an indicator of nutrition, are prognostic for many cancer types [9].

Jung et al. [10] retrospectively analyzed the clinicopathological characteristics of 1,224 operated colon cancer patients and seven blood parameters including albumin (ALB) level, fibrinogen (FBR) level, lymphocyte count (LYM), monocyte count (MON), neutrophil count (NEU), platelet count (PLT), and white blood cell (WBC) count in the four weeks preoperatively. In 2022, they suggested that the Prognostic Immune Nutritional Index (PINI), which is calculated as (ALB × 0.9) - (MON × 0.0007), could be used prognostically in operated colon cancer patients. Our study aimed to research the prognostic significance of this novel prognostic marker in mCC patients.

## Materials and methods

The Ethics Committee of Suleyman Demirel University Faculty of Medicine approved this study on April 1, 2022, with protocol number 8/103. There was no need for scientific research funding because the investigation was retrospective. There is no conflict of interest in this study.

Patients

The patients included in the study applied to the Medical Oncology Unit of Suleyman Demirel University Faculty of Medicine with a histopathological diagnosis of colon cancer with metastasis between 2010 and 2020. Patients over the age of 18 who continued their follow-up in our university Medical Oncology Unit with a diagnosis of metastatic colon cancer and who had necessary information in the hospital’s Picture Archiving and Communication Systems (PACS) and had a metastatic colon cancer diagnosis were included in the study. Patients who did not come to their checkups regularly and did not have the necessary information in the PACS were not included in the study.

The parameter used was calculated as explained in the article. PINI was calculated as follows: (albumin (g/L) × 0.09) - (monocytes × 0.0007).

Statistical methods

The Statistical Package for the Social Sciences (SPSS) version 26.0 (IBM SPSS Statistics, Armonk, NY, USA) was used for statistical analysis, and MedCalc version 20.027 (MedCalc, Ostend, Belgium) was used for receiver operating characteristics (ROC) analysis. Continuous data and discrete data were recorded for descriptive statistics. Descriptive analyses were used for demographic data. The chi-squared test was used to compare the ratios in the groups, and the Kaplan-Meier (log-rank test) method was used to measure and analyze some descriptive indices. The Kaplan-Meier log-rank test assessed the equality of survivorship for the outcome measure of overall survival (OS). The primary objective of the analysis was to identify factors associated with OS. A comparison of the groups in terms of survival was made using the log-rank test. An actuarial study was first done using the Kaplan-Meier technique with two-tailed log-rank p values to investigate potential predictive variables. The p values were two-sided, with 0.05 being considered statistically significant. We built confounder models using the Cox proportional hazards model based on univariate analyses to estimate how patient demographics and clinicopathologic tumor characteristics affected survival. The results provided hazard ratios and 95% confidence intervals (CIs) adjusted for all variables in the model. A “p” value less than 0.05 was considered significant in statistical analyses.

## Results

Patient characteristics

Of 190 patients, 35.6% (n=68) were women and 63.9% (n=122) were men. The mean age of the participants was 62.64±11.99 years. Of tumors, 76.8% (n=146) were in the left colon and 23.2% (n=44) were in the right colon. The median follow-up time was 25.81 months. In our study, the three-year OS rate was 37.3%. The median OS was 32 months (95% CI: 26.08-37.12). According to PINI, the median OS in patients with PINI<3 was 22.70 months (95% CI: 16.05-29.35), and the median OS in patients with PINI≥3 was 38.83 months (95% CI: 26.98-37.01) (p<0.001).

At least 130 (68.4%) people died, and 48 (25.3%) people survived; we did not have any information about 12 (6.3%) patients. Of the patients, 57.4% (n=78) had liver metastasis and 28.7% (n=39) had lung metastasis. When RAS mutation was checked, 41 (21.6%) patients were positive, 39 (20.5%) patients had KRAS mutation, and two (1.1%) patients had NRAS mutation. Forty-four (23.2%) patients had tumors in the right colon, and 146 (76.8%) patients had tumors in the left colon. As seen in Table 1, liver metastasis rates were found to be significantly higher in patients with PINI<3.

**Table 1:** Characteristics of patients *Chi-squared test **Mann-Whitney U test SD: standard deviation, BMI: body mass index

Characteristics	Number (%)	PINI<3	PINI≥3	p
Age, years, mean±SD (range)	62.34±11.99	62.76±10.74	61.93±13.14	0.998**
Gender				
Women	68 (35.8)	31 (33)	37 (38.5)	0.424*
Men	122 (64.2)	63 (67)	59 (61.5)	
BMI, mean±SD	26.79±4.37	26.80±4.74	26.76±4.02	0.816**
Tumor location				
Left colon	146 (75)	74 (78.7)	72 (75)	0.543*
Right colon	44 (25)	20 (21.3)	24 (25)	
Liver metastases				
Yes	78 (57.4)	47 (60.3)	31 (39.7)	0.002*
No	58 (42.6)	22 (37.9)	36 (62.1)	
Lung metastases				
Yes	39 (28.7)	15 (21.7)	24 (35.8)	0.225*
No	97 (71.3)	54 (78.3)	43 (64.2)	
RAS mutation				
Yes	41 (27.2)	23 (24.5)	18 (18.8)	0.338*
No	149 (72.8)	71 (75.5)	78 (81.3)	
Total	190 (100)	94 (100)	96 (100)	

Univariate analysis of prognostic factors for OS

Table 2 lists all seven potential predictive factors along with their univariate analyses. According to univariate log-rank analysis, having liver metastasis and having lower PINI were significantly associated with overall survival (p<0.001) (Table 2). Lower PINI (<3) increased the risk of death 2.1 times compared to patients with higher PINI (≥3) (odds ratio (OR)=2.071, p<0.001). Having liver metastasis increased the risk of mortality 1.4 times compared to patients who did not have liver metastasis (OR=1.429, p=0.049).

**Table 2:** Univariate analysis of baseline characteristics for overall survival HR: hazard ratio, CI: confidence interval, SD: standard deviation, BMI: body mass index, PINI: Prognostic Immune Nutritional Index

Characteristics	Overall survival univariate analysis		
	HR	95% CI	p
Age, years, mean±SD (range)	1.008	0.994-1.023	0.264
Gender			
Women	1		
Men	1.022	0.713-1.466	0.905
BMI, mean±SD	1.004	0.959-1.051	0.879
Tumor location			
Left colon	1		
Right colon	1.165	0.762-1.779	0.481
Liver metastases			
No	1		
Yes	1.429	1.001-2.040	0.049
Lung metastases			
No	1		
Yes	1.404	0.937-2.105	0.100
RAS mutation			
Yes	1.125	0.754-1.680	0.563
No	1		
PINI			
<3	2.095	1.468-2.095	<0.001
≥3	1		

Multivariate analysis of prognostic factors for OS

**Figure 1:**
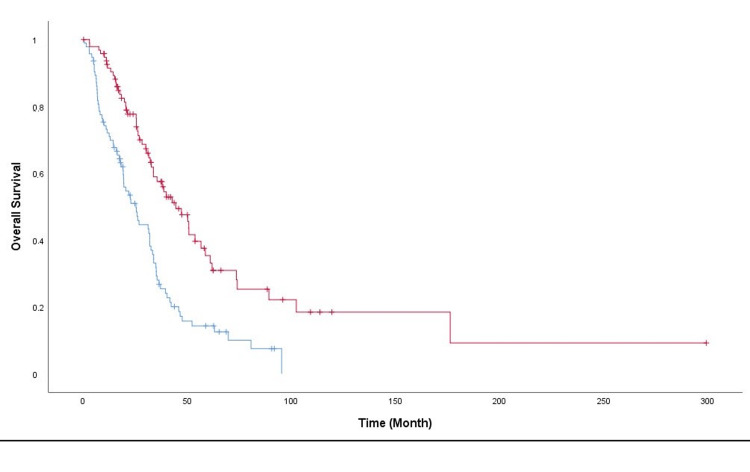
Kaplan-Meier curves for overall survival according to PINI scores PINI: Prognostic Immune Nutritional Index Upper (pink) line: PINI≥3, lower (blue) line: PINI<3

Multivariate analysis revealed that one baseline variable independently predicted OS in metastatic colon patients. PINI was significantly predictive of OS in metastatic colon patients; this was the only prognostic indicator in multivariate analysis (Table 3, Figure 1).

**Table 3:** Multivariate analysis of prognostic factors and log-rank analysis OR: odds ratio, CI: confidence interval, PINI: Prognostic Immune Nutritional Index

Parameter	Number of patients		Median overall survival (months)	Log-rank p value	Multivariate analysis		p value
Gender	Died	Total			OR	95% CI	
Women	17	62	33.40±5.16	0.993	1.162	0.763-1.771	0.483
Men	31	107	30.50±2.68		1		
Tumor location							
Left colon	134	32	32.30±1.65	0.480	1.232	0.728-2.085	0.437
Right colon	44	16	25.60±5.90		1		
Liver metastases							
No	29	78	35.57±4.25	0.048	1.325	0.860-2.041	0.202
Yes	19	100	27.00±2.73		1		
Lung metastases							
No	31	130	30.50±2.63	0.098	1.219	0.742-2.004	0.435
Yes	17	48	37.97±5.97		1		
RAS mutation							
Yes	7	39	26.13±3.06	0.563	1		0.139
No	41	139	32.80±1.51		1.418	0.893-2.251	
PINI							
<3	13	76	22.70±3.39	<0.001	1		<0.001
≥3	35	54	38.83±5.25		2.071	1.377-3.115	

Multivariate analysis revealed that one important value was independently predictive of OS with metastatic colon cancer. PINI lower than 3 was an independent prognostic indicator in multivariate analysis.

## Discussion

The relationship between cancer progression, systemic inflammatory response, and inflammation markers and their effect on malignancy progression and survival have been extensively researched [11]. In oncological malignancies, biochemical and hematological markers were used to assess the impact of the systemic inflammatory response on outcomes such as increased C-reactive protein (CRP) concentration, increased white cell, neutrophil, and platelet counts, and hypoalbuminemia [12]. A chronically inflamed microenvironment can influence cell proliferation, causing cells to lose their ability to control growth, leading to hyperproliferation and tumorigenesis [13]. The inflammatory response is critical in carcinogenesis, and various inflammatory cells and innate immune system signaling molecules, such as neutrophils, lymphocytes, platelets, and monocytes, are involved in tumor progression. As a result, NLR, PNI, PLR, and MLR, which represent a systematic inflammatory response, may be prognostic factors for mCC [14]. Several studies confirmed the potential prognostic utility of NLR in colon cancer patients. NLR was a poor prognostic marker in colon cancer patients undergoing elective surgery [15-18]. Some studies, however, contradict previous ones; for example, NLR was not found to be associated with colon cancer prognosis [19,20].

The PNI is calculated as follows: (10 serum albumin (g/dL)) + (0.005 total lymphocyte count). Several studies found it to be a positively correlated prognostic marker for overall survival in colon cancer patients undergoing surgery [21-24]. It was also determined to be an independent prognostic factor for mCC patients [25]. In multiple studies, hypoalbuminemia was associated with poor OS in mCC and poor disease-free survival in colon cancer patients undergoing surgery [26-28]. In multiple studies, the monocyte count in different prognostic markers was associated with OS in mCC patients [29,30]. PINI was recently proposed as a novel and strong prognostic marker for metastatic colon cancer [10].

As the best prognostic marker, PINI is calculated using albumin and monocyte counts. There was a cutoff point of 3, which was found by Jung et al. [10], so we used the same cutoff in our article. Following the findings of this study, we wondered whether or not our patient values would confirm this hypothesis. Our study found PINI to be an independent prognostic factor in mCC. The median OS was 38.83 months in the PINI≥3 group and 22.7 months in the PINI<3 group (p<0.001). Mortality risk due to mCC was 2.1 times higher in the low PINI group than in the high PINI group. Jung et al. found that the survival of patients with high PINI (≥3) values was better than those with low PINI (<3) values (five-year OS: 84.4% versus 62%, p<0.001). While the patients in Jung et al.’s study were operated colon cancer patients, the patients in this study were metastatic colon cancer patients, so this is the first study in the literature to investigate the prognostic value of PINI in metastatic colon cancer patients. The results of our study are consistent with those of Jung et al. that PINI can be used as a prognostic marker in patients with colon cancer [10]. Due to the study being completed in a single center and the retrospective design, our study has some limitations. However, confirming the suggestion that PINI is a robust prognostic marker for patients with metastatic colon cancer is vital.

## Conclusions

A novel and powerful prognostic marker, PINI, which can be calculated using a simple formula, can provide important clues to clinicians when deciding on individual treatment. More research is needed to support this hypothesis and help clinicians dealing with cancer patients to easily evaluate prognosis and treatment approaches.
